# Bicyclol Alleviates Signs of BDL-Induced Cholestasis by Regulating Bile Acids and Autophagy-Mediated HMGB1/p62/Nrf2 Pathway

**DOI:** 10.3389/fphar.2021.686502

**Published:** 2021-07-21

**Authors:** Jingwen Zhao, Maojuan Ran, Ting Yang, Liwei Chen, Peixu Ji, Xiuxiu Xu, Lu Zhang, Siyuan Sun, Xin Liu, Simin Zhou, Lu Zhou, Jie Zhang

**Affiliations:** ^1^ Department of Gastroenterology and Hepatology, Tianjin Medical University General Hospital, Tianjin Institute of Digestive Disease, Tianjin Key Laboratory of Digestive Diseases, Tianjin, China; ^2^ Department of Gastroenterology and Hepatology, Chengdu Second People’s Hospital, Chengdu, China; ^3^ Department of Gastroenterology, Shanxi Provincial People’s Hospital, Taiyuan, China

**Keywords:** liver injury, cholestasis, BDL, high mobility group box 1, autophagy, Nrf2, bicyclol

## Abstract

Cholestasis is a liver disease characterized by the accumulation of toxic bile salts, bilirubin, and cholesterol, resulting in hepatocellular damage. Recent findings have revealed several key steps of cholestasis liver injury including the toxicity of bile acids and accumulation of proinflammatory mediator. In this study, we investigated the protective effect of bicyclol in cholestasis caused by bile duct ligation (BDL), as well as relevant mechanisms. Bicyclol attenuated liver damage in BDL mice by increasing the levels of hydrophilic bile acid such as α-MCA and β-MCA, regulating bile acid-related pathways and improving histopathological indexes. High-mobility group box 1 (HMGB1) is an extracellular damage-associated molecular pattern molecule which can be used as biomarkers of cells and host defense. Bicyclol treatment decreased extracellular release of HMGB1. In addition, HMGB1 is also involved in regulating autophagy in response to oxidative stress. Bicyclol promoted the lipidation of LC3 (microtubule-associated protein 1 light chain 3)-Ⅱ to activate autophagy. The nuclear factor, E2-related factor 2 (Nrf2) and its antioxidant downstream genes were also activated. Our results indicate that bicyclol is a promising therapeutic strategy for cholestasis by regulating the bile acids and autophagy-mediated HMGB1/p62/Nrf2 pathway.

## Introduction

Cholestasis is a pathological condition caused by obstruction or cessation of bile flow resulting in intrahepatic cholestasis and accompanied by cell injury, inflammatory infiltration, cell apoptosis, liver fibrosis, and even cirrhosis (Yokoda and Carey, 2019). Currently, the main drug treatment for chronic cholestatic liver injury is ursodeoxycholic acid (UDCA). However, some patients do not respond to this treatment (Wagner and Fickert, 2020). Hence, novel treatment approaches, which could be based on a comprehensive understanding of the mechanisms of the different stages of disease progression, are urgently needed.

A previous study found that primary bile acids conjugated with glycine or taurine were significantly increased in serum of patients with cholestasis liver injury (Woolbright et al., 2015). Bile acids (BAs), such as taurocholic acid (TCA), glycocholic acid (GCA), glycochenodeoxycholic acid (GCDCA), and glycodeoxycholic acid (GDCA), can be used as biomarkers of liver injury or as indicators of drug-induced liver injury (DILI) (Yang et al., 2019). The hydrophobicity of bile acids was affected by the number and orientation of the hydrophilic region, where lithocholic acid (LCA) was the most hydrophobic while cholic acid (CA) was less hydrophobic (Ashby et al., 2018). On the other hand, the conjugated groups also affected the hydrophobicity of bile acids, where unconjugated was the most hydrophobic and glycine conjugated was more hydrophobic than taurine or sulphate conjugated (Ashby et al., 2018). The toxicity of bile acids is mostly dependent on its hydrophobicity. According to previous studies, TCA, the major endogenous BAs in rodents, significantly increases the mRNA level of inflammatory cytokines monocyte chemotactic protein 1 (Ccl2) and macrophage inflammatory protein 2α (cxcl2) (Cai et al., 2017). The toxicity of BAs during obstructive cholestatic liver injury likely occurs because of biliary BAs leakage. Previous studies proved that biliary BAs at 0.5x dose resulted in significant hepatocyte necrosis (Woolbright et al., 2015).

High-mobility group box-1 (HMGB1) protein, a member of the high-mobility group 1 (HMG-1) family, modulates cell death in acute liver injury by NF-κB signal pathway activated by advanced glycan end products (RAGE) and toll-like receptor 4 (TLR4) (Eguchi et al., 2014). HMGB1 is involved in many liver diseases. Previous studies have found that the level of liver HMGB1 protein was correlated with alcoholic steatohepatitis (ASH) and primary biliary cirrhosis (PBC) in patients with chronic Hepatitis C virus (HCV), as well as in chronic carbon tetrachloride (CCl4) treated mice (Ge et al., 2018). HMGB1 levels also increased in BDL induced cholestatic mice (Woolbright et al., 2013; Dondorf et al., 2017), while carnosic acid provided protection against BDL induced fibrosis by inhibiting HMGB1 expression (Zhang et al., 2017a). HMGB1 is a proinflammatory cytokine released by injured cells and the innate immune system, which alerts other cells that infection or sterile injury has occurred (Andersson and Tracey, 2011). Neutrophils and Cxcl2, Ccl2 mRNA levels were significantly decreased in mice with hepatocyte-specific Hmgb1 knockout (Huebener et al., 2019).

Autophagy deficiency leads to the rise of HMGB1 levels and promotes ductular reaction and tumorigenesis *via* its receptor RAGE (Khambu et al., 2018). HMGB1 regulates autophagy under the circumstances of oxidative stress (Kang et al., 2011). The oxidation HMGB1 binding to Beclin1 causes the dissociation of Bcl-2 from Beclin1, leading to the induction of autophagy (Tang et al., 2010). So, autophagy and HMGB1 are closely connected. Autophagy includes five processes: initiation, elongation, closure, maturation, and degradation. LC3B and p62 are the most commonly used markers of autophagy. The carboxyl terminus of the LC3 protein is specifically cleaved to form LC3-Ⅰ with the exposed carboxyl-terminal glycine, which is combined with phosphatidylethanolamine to form LC3-Ⅱ (Kuma et al., 2017). LC3-Ⅱ targets the inner and outer membranes of autophagosomes to participate in the elongation step of autophagy (Kuma et al., 2017; Qian et al., 2017). P62 as a receptor for ubiquitinated proteins and organelles is selectively transported into the autophagosomes, after which it is degraded by autophagy (Ichimura and Komatsu, 2010).

The high levels of p62 associated with the suppression of autophagy competitively interacts with Kelch-like ECH-associated protein-1 (Keap1), the nuclear factor Nrf2-binding site, resulting in activation of Nrf2 and its target genes (Komatsu et al., 2010; Jiang et al., 2015). The activation of Nrf2 protects from various liver diseases through different molecular mechanisms (Xu et al., 2018). After activation, Nrf2 is translocated to the nucleus to activate heme oxygenase-1 (HO-1), NADPH quinone oxidoreductase-1 (NQO1), glutamate cysteine ligase (GCLC), and other genes to resist damage caused by oxidative stress (Galicia-Moreno et al., 2020). Nrf2-knockout mice are more susceptible to hepatic injury after BDL, as vitamin A activates Nrf2 to protect liver function in BDL rats, thus proving that Nrf2 activation is beneficial in cholestatic liver injury (Weerachayaphorn et al., 2012; Shin et al., 2013; Wang et al., 2014).

Bicyclol (4,4′-dimethoxy-5,6,5′,6′-bis (methylenedioxy)-2-hydroxymethyl-2′-methoxycarbonyl biphenyl) is a synthetic drug widely used to treat HCV (Li et al., 2018; Huang et al., 2019). Bicyclol has been proven to induce autophagy and inhibit cell proliferation through PI3K/AKT and MEK/ERK pathways (Wang et al., 2016b). Previous studies in our laboratory have shown that bicyclol can activate Nrf2 to act against CCl_4_ induced hepatotoxicity (Zhao et al., 2020). What’s more, Zhen, Yong-Zhan et al. have found that BDL-induced liver fibrosis can be significantly attenuated by bicyclol through reversing fibrogenic gene expression (Zhen et al., 2015). Nonetheless, there are still few studies on the mechanism of the protective effect of bicyclol in BDL-induced liver damage.

In this study, we aimed to explore the protective effect of bicyclol in BDL induced liver injury. Our research was mainly based on two aspects: the synthesis, excretion, and composition of bile acid, and the pathway associated with autophagy-mediated by HMGB1.

## Materials and Methods

### Animals

Eleven-weeks old male (22–23 g) c57BL/6 mice were purchased from Beijing HFK Bioscience CO., LTD. All mice were housed in the SPF animal room with a constant temperature (22°C), and a 12/12 h light/dark cycles and were given free access to water and food. Before the experiment, mice were allowed to adapt to the environment for 1 week. All animal studies (including the mice euthanasia procedure) were done in compliance with the regulations and guidelines of the Animal Ethical and Welfare Committee (AEWC) at the Institute of Radiation Medicine Chinese Academy of Medical Sciences and conducted according to the AAALAC and the IACUC guidelines.

The mice were randomly divided into three groups: Sham group (n = 8), BDL group (n = 6), and BDL + Bicyclol group (n = 6); the different numbers in different groups are due to the injury following BDL surgery. BDL was employed to induce cholestatic liver injury as described in a previous study (Fickert et al., 2002). Briefly, after anesthesia with isoflurane, the bile duct was separated from the portal vein and hepatic artery, then ligated using a 6–0 suture. The sham group underwent similar laparotomy without BDL. BDL + Bicyclol group was orally administered with bicyclol at a dose of 100 mg/kg (Hu and Liu, 2006; Zhen et al., 2015) body weight daily for 14 days, while sham and BDL group were treated with volume-matched 0.5% carboxymethylcellulose sodium (CMC-Na). Bicyclol was a kind gift from the Beijing Union Pharmaceutical Company (Beijing, China) with a purity of over 99%. Animals were sacrificed on days 14 after the operation, after which the blood and liver were collected and stored at −80°C for further research.

### Serum Biochemical Analysis

The serum levels of aspartate transaminase (AST), alanine transaminase (ALT), alkaline phosphatase (ALP), γ-glutamyl transpeptadase (GGT), serum bile acid (TBA), and total cholesterol (TC) were measured by the automated chemistry analyzer (AU5800, Beckman Coulter, United States) of the clinical laboratory (Tianjin medical university general hospital).

### Histology

Liver tissue was fixed in 4% paraformaldehyde at room temperature and blocked with paraffin wax, after which they were sectioned into 4 um thick slices and stained with hematoxylin and eosin (H&E) according to the standard H&E protocol. The inflammatory cell infiltration and necrosis were assessed by two experienced pathologists according to Ishak scoring criteria, including piece-meal necrosis, fusion necrosis, lytic necrosis, and portal inflammation (Ishak et al., 1995). Images were taken by Leica microscope (Leica DM5000B; Germany) at 200X magnification.

### Measurement of Gallbladder Bile Acids Composition

Gallbladder bile was collected, and its composition was measured with liquid chromatography-tandem mass spectrometry (LC-MS/MS) (Yang et al., 2017). Briefly, standard samples were added into 50 ul bile samples, the supernatant was extracted after oscillation and centrifuged at 12,000 rpm for 10 min and then further concentrated on the concentrator. The concentrate was reconstituted in 100 uL of methyl/water (50/50) and analyzed by the LC-MS/MS system. The chromatographic separation was performed on the ACQUITY UPLC HSS T3 column (2.1*100 mm, 1.8 um). Data acquisition systems mainly included Ultra Performance Liquid Chromatography (Shim-pack UFLC SHIMADZU CBM30A) and Tandem mass spectrometry (Applied Biosystems 6500 QTRAP).

### Immunofluorescence

The slides with liver samples were dewaxed with xylene and various ethanol concentrations and blocked with 5% bovine serum albumin (BSA) for 30 min. After that, the slides were incubated with primary antibodies against p62/SQSTM1 (no. NBP1-48320SS; Novus) at 4°C overnight. After being washed with PBS, the sections were incubated with secondary antibodies at 37°C for 1 h. The nuclei were stained with 4′-6′-diamino-2-phenylindole dihydrochloride (DAPI), and then sealed with resins. The sections were observed under a fluorescence microscope (Leica DM5000B; Germany).

### Cell Culture and Treatment

Alpha mouse liver 12 cell line AML12 were purchased from the Chinese Academy of Sciences Cell Bank (Shanghai, China) and cultured in Dulbecco's Modified Eagle Media: Nutrient Mixture F-12 (DMEM—F12) (#119435; Gibco) supplemented with 10% fetal bovine serum (Gibco), 1% Insulin—Transferrin-Selenium (ITS) (#41400045; Gibco),40 ng/ml dexamethasone. Cells were grown in a humidified atmosphere with 5% CO_2_ at 37°C. Once the cells reached confluency near 80%, they were digested with 0.125% trypsin-EDTA and seeded into 6-well plates. For analysis of protein and mRNA levels, cells were stimulated for 24 h with 100 uM TCA and/or 500 uM bicyclol. Taurocholic acid (TCA) was purchased from Sigma-Aldrich.

### Western Blot

Liver tissues and cells were homogenized in RIPA buffer (Solarbio, China) that contained PMSF (Solarbio, China) and phosphatase inhibitors on ice. The homogenates were centrifuged, and supernatants were extracted, after which 1/3 volume of loading buffer was added and stored at −80°C for further study. The protein was quantified by a BCA protein assay kit (Solarbio, China). Equal amounts of proteins were separated by SDS-PAGE gels and transferred to nitrocellulose membranes. After blocking with 5% non-fat milk, membranes were incubated with primary antibodies overnight at 4°C. The primary anti-CYP7A1 (#sc-518007, Santa Cruz), anti-BSEP (#sc-74500, Santa Cruz), anti-FXR (#ab235094, Abcam), anti- SQSTM1/p62 (#39749, Cell Signaling Technology), anti-LC3A/B (D11) (#3868, Cell Signaling Technology), anti-Beclin-1 (#3495, Cell Signaling Technology), anti-HMGB1 (#6893, Cell Signaling Technology), anti-RAGE (ab216329, Abcam, Cambridge, MA, United States), anti-TLR4 (#A17436, ABclonal Technology), anti-NRF2 (#12721, Cell Signaling Technology), anti-Keap1 (#A17061, ABclonal Technology) and anti-β-actin (#3700, Cell Signaling Technology) were used. The secondary horseradish peroxidase-conjugated goat anti-rabbit or anti-mouse IgG antibodies (Zhongshan Golden Bridge Biotechnology, Beijing, China) were incubated at room temperature for 1 h. After fully washing, the ECL Reagent (Solarbio, China) and Image Lab (Bio-Rad, America) were used to analyze the bands.

### Real-Time RT-PCR

Total RNA was extracted from liver tissues, and AML12 cells using Trizol reagent and the concentration was determined by an enzyme-labeled instrument. The RNA was reverse-transcribed into cDNA using SuperScript III First-Strand Synthesis System (Invitrogen, Carlsbad, CA, United States). The reaction mix was performed as follows: 50°C (2 min); 95°C (5 min); followed by 50 cycles of 95°C (15 s) and 60°C (30 s). Relative expression of target genes was calculated by the 2-^ΔΔCt^ method and normalized to GAPDH. The primer sequences are shown in Table 1.

**TABLE 1:** Specific qPCR primers used in this study.

Gene name	Forward primer (5‘-3’)	Reverse primer (5‘-3’)
*Gapdh*	GGA​GAA​ACC​TGC​CAA​GTA​TG	TGG​GAG​TTG​CTG​TTG​AAG​TC
*Cyp7a1*	AAC​AAC​CTG​CCA​GTA​CTA​GAT​AG	GTG​TAG​AGT​GAA​GTC​CTC​CTT​AGC
*Fxr*	GGC​CTC​TGG​GTA​CCA​CTA​CA	TGT​ACA​CGG​CGT​TCT​TGG​TA
*Bsep*	CTG​CCA​AGG​ATG​CTA​ATG​CA	CGA​TGG​CTA​CCC​TTT​GCT​TCT
*Tnf-α*	GGT​GCC​ATG​TCT​CAG​CCT​CTT	GCC​ATA​GAA​CTG​ATG​AGA​GGG​AG
*IL-1β*	GCC​ATA​GAA​CTG​ATG​AGA​GGG​AG	GCC​ATA​GAA​CTG​ATG​AGA​GGG​AG
*Nrf2*	CTT​TAG​TCA​GCG​ACA​GAA​GGA​C	AGG​CAT​CTT​GTT​TGG​GAA​TGT​G
*Nqo1*	AGG​ATG​GGA​GGT​ACT​CGA​ATC	TGC​TAG​AGA​TGA​CTC​GGA​AGG
*Hmox1*	GAT​AGA​GCG​CAA​CAA​GCA​GAA	CAG​TGA​GGC​CCA​TAC​CAG​AAG

### Statistical Analysis

Data are expressed as mean ± SEM and analyzed with GraphPad Prism 8.0.1 (GraphPad Software, United States). One-way ANOVA followed by Tukey’s multiple comparisons test was used to calculate the statistical significance. A *p*-value of <0.05 was considered statistically significant.

## Results

### Protective Effects of Bicyclol on Liver Injury in Bile Duct Ligation Mice

**FIGURE 1:**
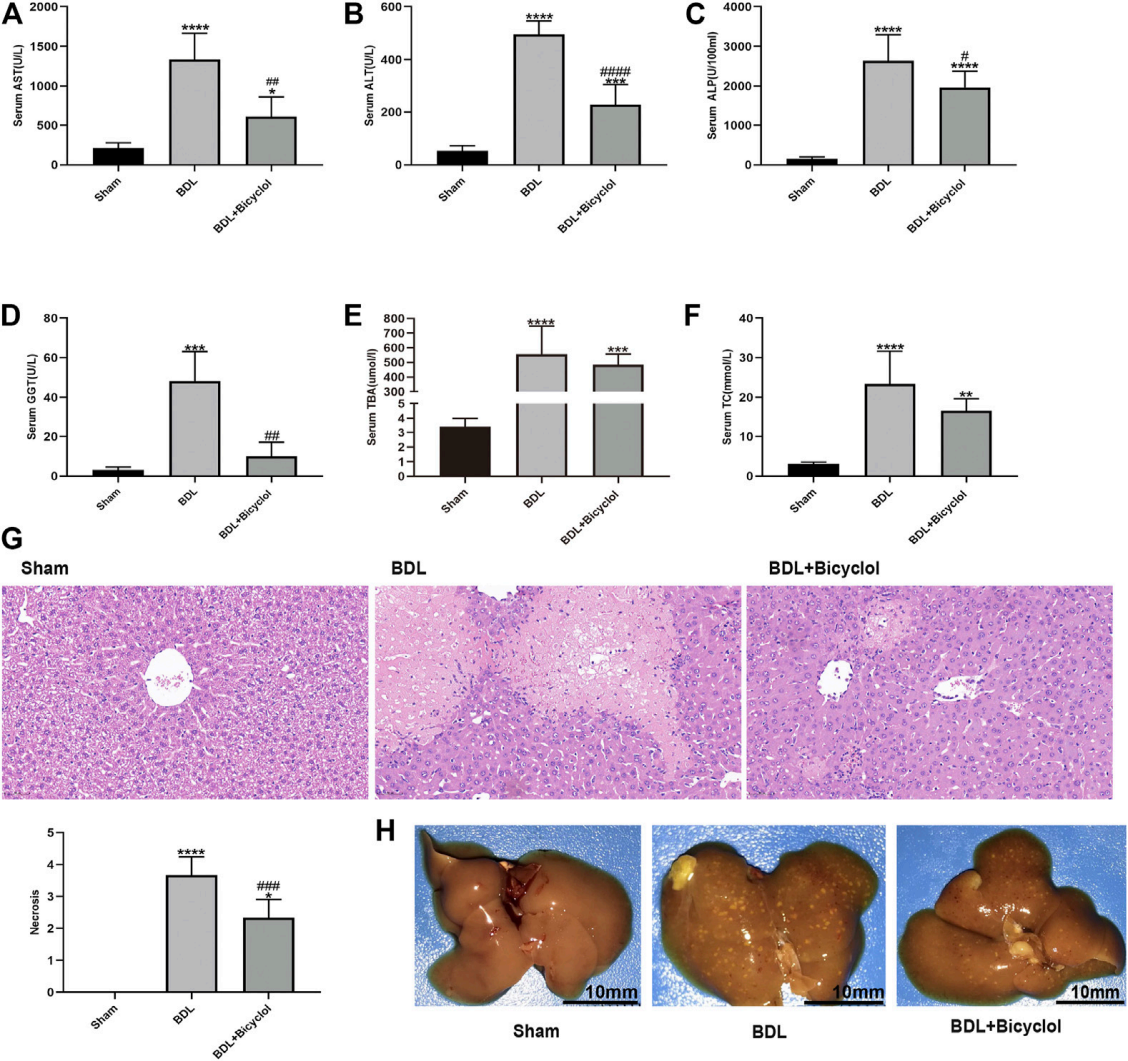
Bicyclol markedly improved the liver enzyme, histology, and liver gross appearance in BDL mice. **(A–F)** Serum biochemistry including AST, ALT, ALP, GGT, TBA, and TC. **(G)** H&E stain liver histology (Scale bar, 100 um). **(H)** Gross liver appearance (Scale bar, 10 mm). AST, aspartate aminotransferase; ALT, alanine aminotransferase; ALP, alkaline phosphatase; GGT, γ-glutamyl transpeptidase; TBA, total bile acid; TC, total cholesterol. *p < 0.05, **p < 0.01, ***p < 0.001, ****p < 0.0001 vs. sham; ^#^ *p* < 0.05, ^##^ *p* < 0.01, ^###^ *p* < 0.001, ^####^ *p* < 0.0001 vs. BDL.

To explore the effects of bicyclol on BDL induced mice liver injury, we tested liver enzymes including AST, ALT, ALP, GGT, TBA, and TC, and liver pathology. The serum AST, ALT, ALP, GGT, TBA, and TC levels increased in BDL compared with the sham group (Figures 1A–F). Bicyclol significantly decreased the AST, ALT, ALP, and GGT levels. However, the TBA and TC levels were comparable between BDL and BDL + Bicyclol groups. As indicated by H&E staining, BDL induced inflammatory cell infiltration and hepatic necrosis in mice liver, the effects of which were then ameliorated by bicyclol (Figure 1G). The beneficial effects were also proved by liver appearance, where livers from BDL mice had many yellow-white granules on the surface. In contrast, yellow-white granules of the liver were significantly improved in the bicyclol treatment group (Figure 1H). These results revealed that bicyclol could relieve BDL induced liver injury.

### Bicyclol Treatment Changed Bile Acid Composition

**FIGURE 2:**
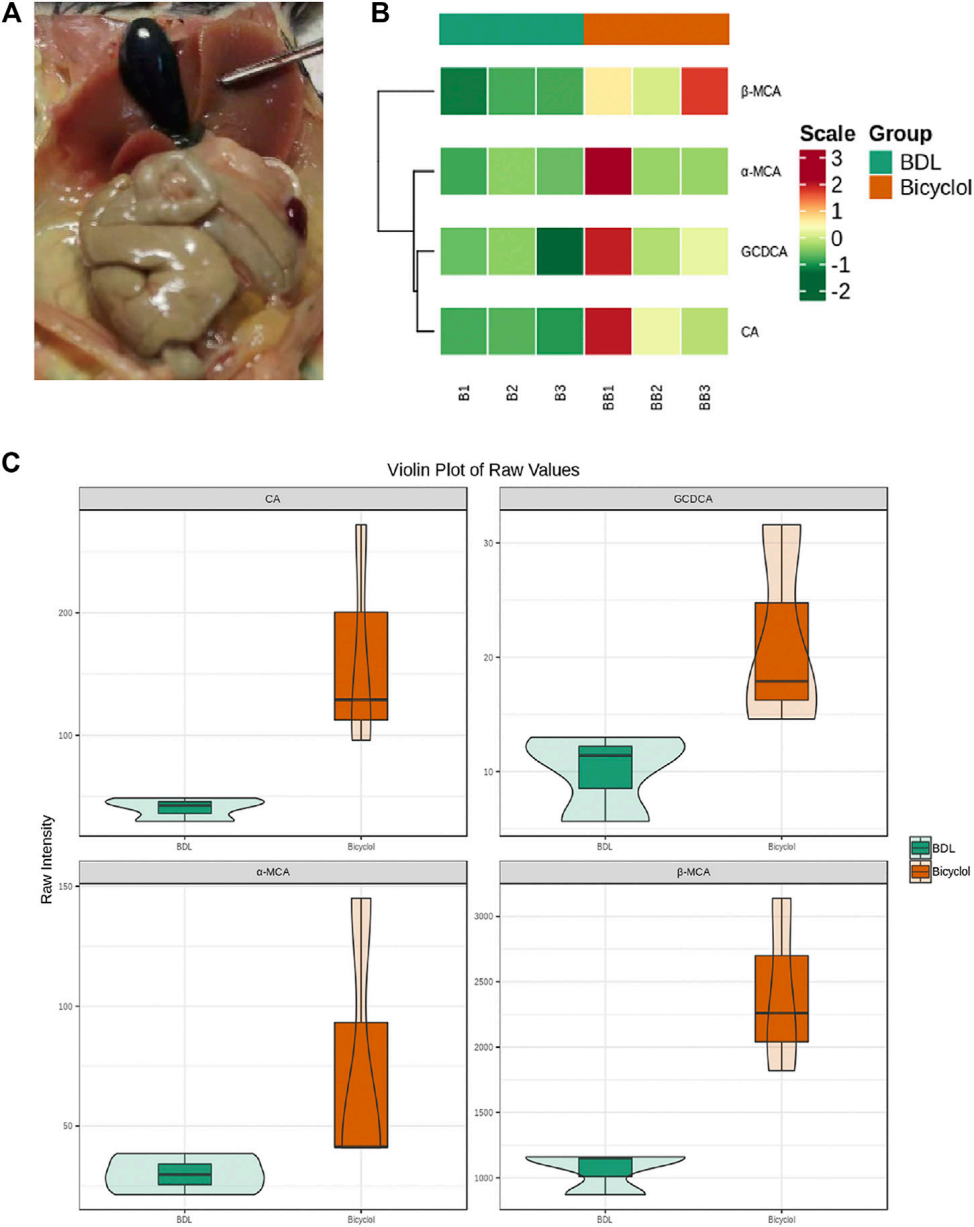
Bile acid composition in the gallbladder. **(A)** The gallbladder of BDL mice. **(B)** Heatmap of differential bile acids of gallbladder bile samples. Rows represent different bile acids, while columns represent different samples. Colors from green to red indicate increasing levels from low to high. **(C)** Violin plot analysis comparing the levels of CA, GCDCA, α-MCA, β-MCA in the BDL and Bicyclol groups. Green represents the BDL group, whereas orange represents the Bicyclol group.

In those mice with bile duct ligation, the gallbladder volume significantly increased, and the bile was stagnant (Figure 2A); thus, we collected the bile in the gallbladder and analyzed the composition. In total, we tested 25 different kinds of bile acids, finding four BAs CA, GCDCA, α-muricholic acid (α-MCA), β-muricholic acid (β-MCA) levels that differed between BDL and bicyclol groups (Figure 2). A heatmap was plotted to show the levels of four bile acids (Figure 2B). CA, GCDCA, α-MCA, β-MCA in three samples of the bicyclol group were higher than that in the BDL group. In addition, violin plot analysis showed that the levels of bile acids, including CA, GCDCA, α-MCA, and β-MCA were higher in the bicyclol group than in the BDL group (Figure 2C). Previous studies have found that cholate and β-muricholate were the main components of mouse bile acid pool (Kerr et al., 2002), with the following order from hydrophobic to hydrophilic: LCA > deoxycholic acid (DCA) > chenodeoxycholic acid (CDCA) > CA > UDCA > MCA (de Aguiar Vallim et al., 2013). Our results showed that bicyclol could increase hydrophilic bile acid, mainly the α-MCA, β-MCA levels.

### Bicyclol Prevented Liver Injury by Preventing Hepatic Bile Acid Synthesis and Promoting Bile Acid Excretion

**FIGURE 3:**
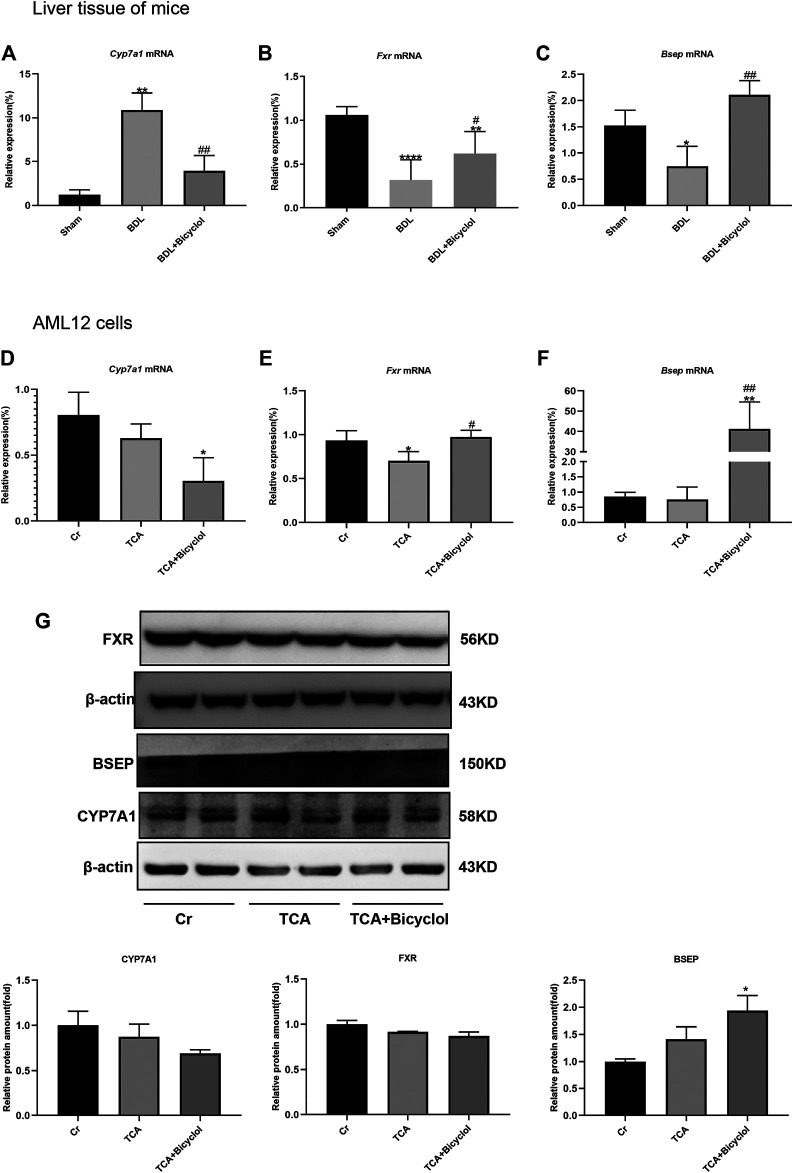
Effects of bicyclol on genes related to bile metabolism. **(A–C)** The mRNA levels of bile metabolism-related genes (Fxr, Cyp7a1, Bsep) in BDL and bicyclol treatment mice. The mRNA levels **(D–F)** and protein levels **(G)** of bile metabolism-related genes (Fxr, Cyp7a1, Bsep) in AML12 cells after TCA and bicyclol exposure. Cr represents control group without special treatment, TCA means cells were stimulated with 100 uM TCA for 24 h, TCA + Bicyclol means cells were pretreated with 500 uM bicyclol for 2 h, then stimulated with 100 uM TCA for 24 h. Data are expressed as mean ± SD. *p < 0.05, **p < 0.01, ***p < 0.001, ****p < 0.0001 vs. sham or Cr; ^*#*^ *p* < 0.05, ^##^ *p* < 0.01 vs. BDL or TCA.

*Cyp7a1* is the key enzyme in the classic bile acid synthesis pathway (Chiang and Ferrell, 2019). *Fxr* is a nuclear receptor that suppresses *Cyp7a1* expression (Xu et al., 2016). In our study, liver *Cyp7a1* mRNA expression level was elevated by BDL and significantly reduced post bicyclol treatment (Figure 3A). BDL significantly reduced *Fxr* expression at the mRNA level, which was then reversed by bicyclol (Figure 3B). *Bsep* expression at the mRNA level was also decreased in the BDL group and increased in BDL + Bicyclol group (Figure 3C). TCA resulted in the most abundant BAs in blood, which was significantly increased in cirrhosis and hepatocellular carcinoma (HCC) patients, and induced inflammatory gene expression in liver cells. Consequently, we treated TCA cells to mimic the cell damage caused by bile (Chen et al., 2011; Wang et al., 2016a). TCA caused a down-regulation of *Fxr* [F (2,7) = 7.736], which was reversed by bicyclol. *Fxr* further caused a decrease of *Cyp7a1* and an increase of *Bsep* (Figures 3D–F). At the same time, bicyclol significantly up-regulated the protein expression of BSEP and inhibited the protein level of CYP7A1 although there was no significant difference, even if the FXR protein expression was not changed by bicyclol (Figure 3G). These results suggested that bicyclol could activate *Fxr* to suppress *Cyp7a1* expression, which led to a reduction of BA synthesis and acceleration of BA excretion.

### Bicyclol Ameliorates Bile Duct Ligation-Induced Liver Injury by Down-Regulation of High-Mobility Group Box-1

**FIGURE 4:**
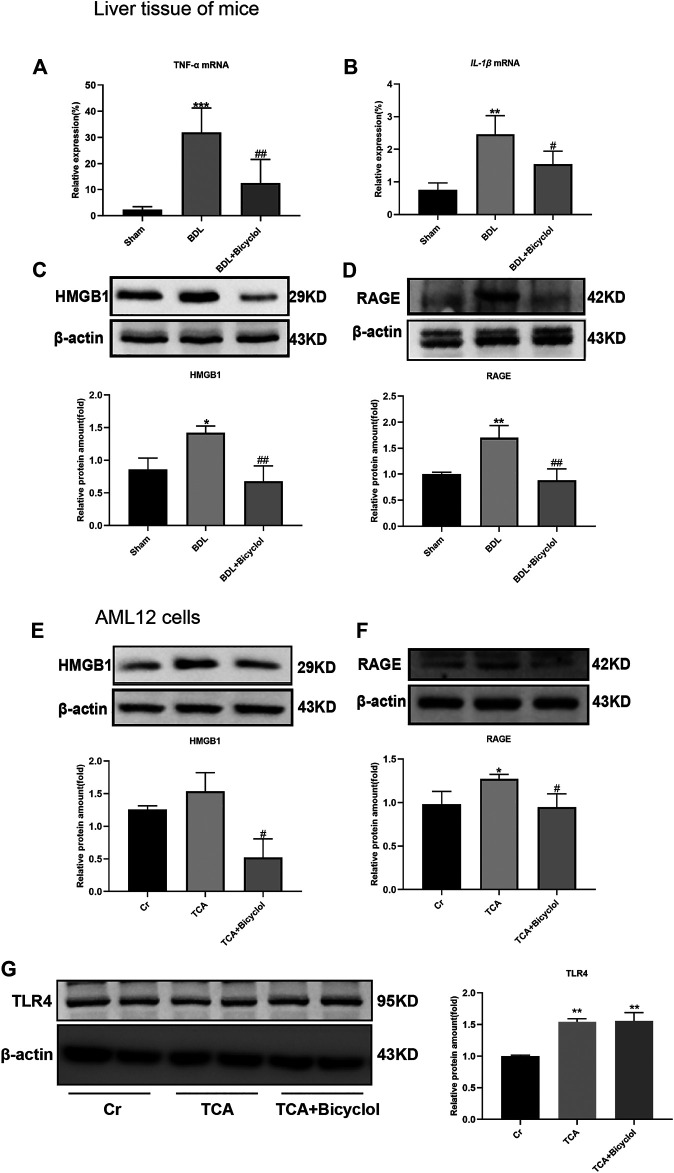
Effect of bicyclol on inflammatory cytokines, HMGB1, and its receptor RAGE and TLR4. The mRNA level of Tnf-α **(A)** and IL-1β **(B)** in mouse liver tissue. **(C,D)** Immunoblots for HMGB1 and RAGE in mouse liver. **(E,F)** Immunoblots for HMGB1 and RAGE in AML12 cells. **(G)** Immunoblots for TLR4 in AML12 cells. *p < 0.05, **p < 0.01, ***p < 0.001 vs. sham or Cr; ^#^ *p* < 0.05, ^##^ *p* < 0.01 vs. BDL or TCA. Protein levels were normalized to levels of β-actin.

Inflammatory factors, including tumor necrosis factor-α (TNF-α), interleukin-1 β (IL-1β), interleukin- 6 (IL-6), transforming growth factor-β (TGF-β), are involved in drug-induced liver injury, cholestasis, alcoholic and non-alcoholic fatty liver diseases, and other chronic liver disease processes (Szabo and Csak, 2012). Severe bile duct damage often triggers inflammation. To further verify the liver damage caused by BDL and the effect of bicyclol, we tested the mRNA levels of *TNF-α* and *IL-1β*. The results showed that bicyclol recovered the BDL-induced *IL-1β*, *TNF-α* mRNA higher expression (Figures 4A,B). HMGB1, as a member of damage-associated molecular patterns (DAMPs) and its receptor RAGE, promote neutrophil infiltration in necrotic tissue, thus aggravating necrosis (Huebener et al., 2015). So, we also measured the protein expression changes of HMGB1 in the liver and AML12 cells. The HMGB1 expression levels were upregulated in the BDL or TCA groups, and this upregulation was offset by bicyclol (Figures 4C,E **)**. Similarly, BDL and TCA also caused the up-regulation of RAGE protein and was also reversed by bicyclol (Figures 4D,F). TLR4 can be activated by HMGB1, the expression of TLR4 was upregulated at TCA and TCA + Bicyclol groups compare to Cr group (Figure 4G).

### Bicyclol Stimulated Autophagy in Mice and Alpha Mouse Liver 12 Cells

**FIGURE 5:**
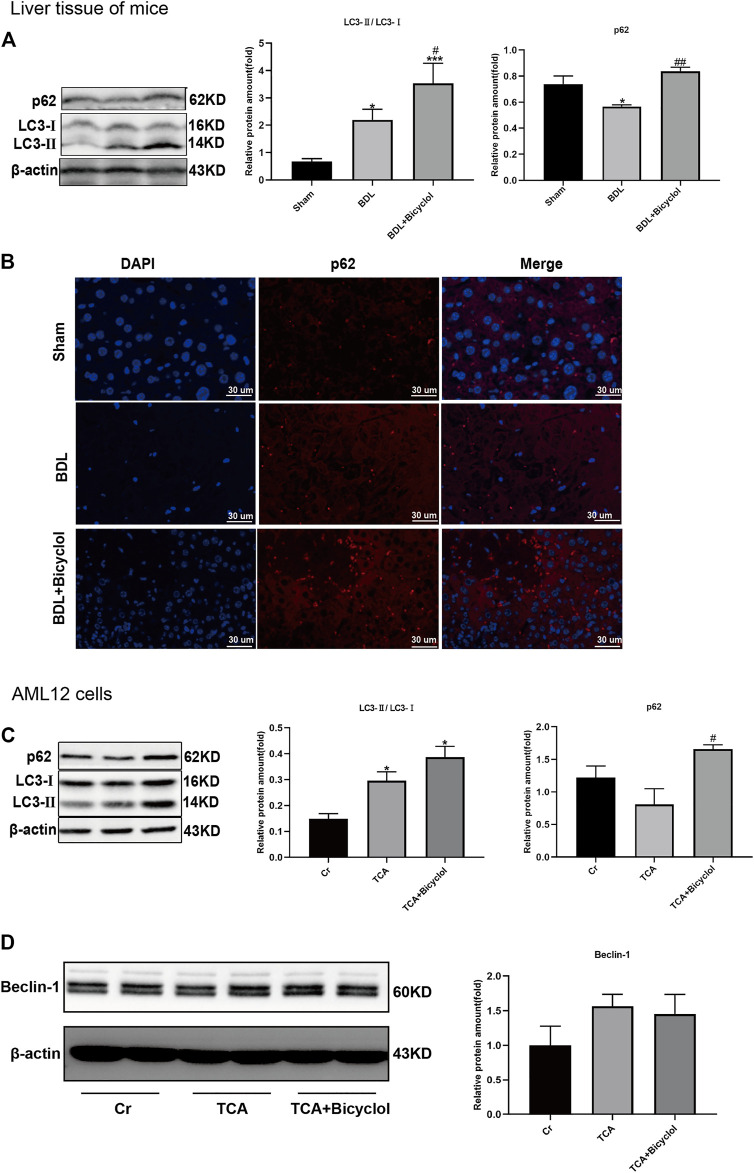
Effect of bicyclol on autophagy. **(A)** P62 and LC3-Ⅱ protein levels in mice were detected by western blot. **(B)** Paraffin sections of liver tissue were stained with p62 (red) and counterstained with DAPI to visualize nuclei (blue) by immunofluorescence. Scale bar, 30 um. **(C)** Immunoblots for p62 and LC3-Ⅱ in AML12 cells. **(D)** Immunoblots for Beclin-1 in AML12 cells. *p < 0.05 vs. sham or Cr; ^#^ *p* < 0.05, ^##^ *p* < 0.01, ^###^ *p* < 0.001 vs. BDL or TCA. Protein levels were normalized to levels of β-actin.

To analyze the effect of bicyclol on autophagy, two important markers of autophagy were detected. The conversion from LC3-Ⅰ (soluble form) to LC3-Ⅱ (lapidated form) and p62 protein are autophagy activation indicators. Western blot showed that the expression of LC3-Ⅱ levels was increased, and p62 was decreased in the BDL group. Also, bicyclol treatment further upregulated the LC3-Ⅱ., but the p62 levels were also increased by bicyclol (Figure 5A). *In vitro*, bicyclol enhanced the TCA induced accumulation of LC3-Ⅱ and p62 (Figure 5C). We also observed the expression of p62 in the liver by immunofluorescence (Figure 5B). The results showed that the nucleus of the cholestasis site was broken and dissolved, there was no normal structure, and the fluorescence of p62 was significantly reduced. Bicyclol significantly improved cell necrosis with p62 fluorescence increased dramatically (Figure 5B). These results were consistent with the western blot findings. Besides these, the expression levels of pro-autophagy protein Beclin-1 were upregulated by TCA, even though there was no significant difference (Figure 5D).

### Bicyclol Regulated Nuclear Factor, E2-Related Factor 2 Mediated Antioxidant Response

**FIGURE 6:**
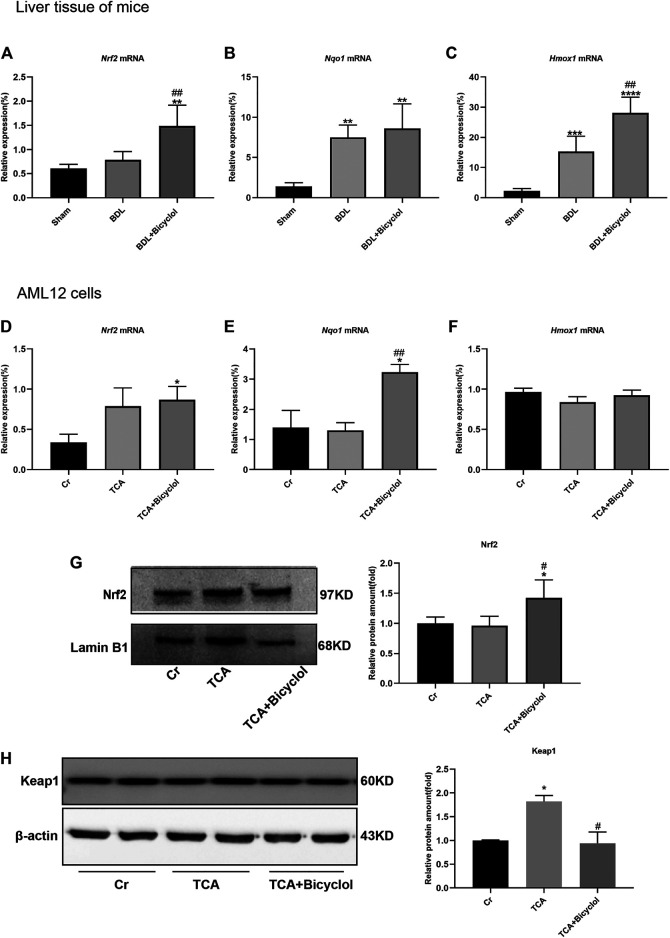
Effects of bicyclol on Nrf2 and its target genes. **(A–C)** The mRNA levels of Nrf2, Nqo1, Hmox1 in BDL and bicyclol treatment mice. **(D–F)** The mRNA levels of Nrf2, Nqo1, Hmox1; The protein levels of Nrf2 **(G)** and Keap1 **(H)** in AML12 cells after TCA and bicyclol exposure. Dates are expressed as mean ± SD. *p < 0.05, **p < 0.01, ***p < 0.001, ****p < 0.0001 vs. sham or Cr; ^#^ *p* < 0.05, ^##^ *p* < 0.01 vs. BDL or TCA.

Cholestasis is related to oxidative stress. Under basal conditions, Nrf2 binds to Keap1 in the cytoplasm; under oxidative stress, Nrf2 dissociates from the Nrf2/Keap1 complex and translocate to the nucleus, thereby initiating the transcription of anti-oxidative stress genes. As shown in Figure 6A *Nrf2* mRNA level was significantly increased in BDL + Bicyclol group compared with the Sham and BDL groups. In addition, the downstream target genes, including NADPH: quinone oxidoreductase 1 (*Nqo1*) and heme oxygenase-1 (*Hmox1*), were also determined. The results showed that *Nqo1* and *Hmox1* were upregulated in BDL and BDL + Bicyclol groups compared with the Sham group (Figures 6B,C). The level of *Hmox1* was further upregulated by bicyclol. The *Nrf2* and *Nqo1* mRNA levels were increased in bicyclol-treated AML12 cells; however, there were no significant differences in TCA-treated AML12 cells (Figures 6D,E). In addition, the protein level of Nrf2 also increased by bicyclol (Figure 6G). The expression level of Keap1 was significant increased after TCA exposure, while pretreatment with bicyclol reversed the upregulation (Figure 6H).

## Discussion

Cholestasis is a liver disease characterized by the accumulation of toxic bile salts, bilirubin, and cholesterol, resulting in hepatocellular injury, which eventually develop into fibrosis and cirrhosis. UDCA, as an exogenous hydrophilic bile acid that replaces endogenous hydrophobic bile acids and obeticholic acid as FXR agonists, have been used to treat PBC (Gulamhusein and Hirschfield, 2020). Besides UDCA and obeticholic acid, 24-norursodeoxycholic acid (norUDCA) is also used to treat PSC and cholestasis liver diseases with favorable outcomes (Fickert et al., 2013; Fickert et al., 2017). Unfortunately, 40% of patients do not have a satisfactory response to UDCA and are at risk of cirrhosis or non-neoplastic complications (Leung et al., 2020; Wagner and Fickert, 2020). The results of the present study demonstrated that BDL could lead to the accumulation of serum TBA and TC which induce a significant liver injury, as evidenced by the increasing of serum ALT, AST, ALP, GGT and hepatic necrosis. Bicyclol treatment exhibited significant protection against BDL induced liver injury by reducing the elevated serum ALT, AST, ALP, GGT levels and hepatic necrosis. Bicyclol also improved bile stasis in the liver as evidenced by the liver gross appearance that yellow-white granules were significantly decreased by bicyclol. These results are consistent with a previous study that bicyclol treatment ameliorated liver condition in BDL rats (Zhen et al., 2015). In addition, our research supplements the potential mechanisms that may be involved.

The composition of bile acids in different species is different. In the human body, CA and CDCA are the main components, while CA and muricholic acid (MCA) are the main bile acid components in mice (de Aguiar Vallim et al., 2013). Due to the presence or absence of hydroxyl groups in the corresponding position and direction on the molecular skeleton, bile acids’ hydrophilicity is different. The order from hydrophilic to hydrophobic reads as follows: MCA > UDCA > CA > CDCA > DCA > LCA (de Aguiar Vallim et al., 2013). The previous study has shown that colesevelam can improve cholestatic liver and biliary tract damage by changing biliary BA composition, mainly by causing hydrophilization of BAs (up-regulating liver and biliary α-MCA, β-MCA and ω-MCA levels) (Fuchs et al., 2018). Another study found that the FXR agonist obeticholic acid increased the levels of α/β MCA from 11.3 to 29.4% and reduced the absorption of cholesterol (Xu et al., 2016). Our experiments revealed that the levels of hydrophilic bile acids such as α-MCA and β-MCA were significantly up-regulated after bicyclol treatment compared to the BDL group. It seems that bicyclol treatment promoted the excretion of the bile acids from the body through the circulation, thus reducing the toxic damage of bile acids. Clinical trials have also found that the bile acid composition in PBC patients treated with UDCA was also changed as the bile was enriched with UDCA, while CA and CDCA were significantly down-regulated, and endogenous bile acids combined with glycine were also increased (Combes et al., 1999). Yet, in our study, we found a different result, i.e., bicyclol treatment increased the levels of CA and GCDCA. Our explanation is that while bicyclol improves the laboratory indicators of BDL accompanied by ongoing liver damage, but GCDCA accumulation can cause liver cell damage because of its hydrophobicity.

BAs are synthesized from cholesterol in the liver through classical and alternative pathways that involve 14 enzymes. Among them, the classical pathway is catalyzed by *Cyp7a1* to synthesize primary bile acids CA and CDCA (Russell, 2003). Cyp7a1 overexpressed mice have increased bile acid pool and a higher level of hepatic cholesterol (Li et al., 2011). Nuclear receptors are also involved in BAs balance. *Fxr* regulates most of the bile acid formation processes, including transport and detoxification, and limits the overload of bile acids in liver cells (Wagner et al., 2011). *Fxr* participates in BAs balance through a variety of ways, including inhibiting liver *Cyp7a1* transcription, inducing intestinal *Fgf-15* to activate liver FGF receptor 4 (FGFR-4), repressing Na/taurocholate cotransporter (NTCP), activating the bile salt export pump *Bsep* and similar (Wagner et al., 2011). The Fxr agonist obeticholic acid (OCA) inhibits the expression of *Cyp7a1* by activating liver *Fxr* and *Shp* expression and changes the bile acid pool size and composition to inhibit intestinal cholesterol absorption (Xu et al., 2016). Our results showed that treatment with bicyclol in BDL mice up-regulated *Fxr* transcription while down-regulating *Cyp7a1* and up-regulating the expression of *Bsep*. Many previous studies have proven that gut microbes are involved in the synthesis and excretion of bile acids and the conversion of components, such as VSL#3 probiotics (including eight different probiotic strains) (Degirolamo et al., 2014) and probiotic *Lactobacillus rhamnosus* GG (LGG) (Liu et al., 2020), while bile acids can also affect bacterial structure *in vitro* and *in vivo* (Tian et al., 2020). Besides affecting the growth of intestinal bacteria, bile also participates in anti-adhesion and neutralizing endotoxins (Wiest et al., 2014). The lack of intestinal bile leads to bacterial translocation and intestinal inflammation, which promotes the progression and development of colorectal cancer (Jia et al., 2018). Therefore, intestinal bacteria and pathological changes after BDL should also be investigated due to the lack of bile in the intestinal.

HMGB1 can have both favorable and unfavorable consequences. On the one hand, hepatocyte-specific HMGB1 knockout mice suffer from increased mitochondrial damage and cell death during liver ischemia/reperfusion (Huang et al., 2014). On the other hand, hepatocyte HMGB1 is involved in the pathogenesis of ALD (Ge et al., 2014). Zhang *et al* found that NF-KBp65 was significantly up-regulated after treatment with FXR siRNA or inhibitor, accompanied by the up-regulation of IL-1β and TNF-α (Zhang et al., 2017b). In the present study, we found that bicyclol increased the FXR expression while it decreased the mRNA levels of inflammatory factors TNFα and IL-1β and the protein level of HMGB1 in the liver. Autophagy, which maintains liver homeostasis by eliminating toxic substances in the liver, is involved in accumulation disorders, non-alcoholic fatty liver disease, fibrosis, hepatocellular carcinoma, hyperammonaemia, viral infections and other liver diseases (Hazari et al., 2020). HMGB1 can stimulate autophagy in a variety of ways, including BECN1 in the cytoplasm, HSPB1 in the nucleus, and RAGE outside the cell. Previous studies have also shown that HMGB1 deficiency has no adverse impact on liver function, nor does it affect mitochondrial quality and autophagy (Huebener et al., 2014; Sun and Tang, 2014). We detected LC3-II/I and p62, which are two markers of autophagy. The results showed that BDL resulted in the up-regulation of LC3-II/I ratio and the degradation of p62 to activate autophagy and protect the liver from cholestasis damage. Bicyclol further increased the LC3-II/I ratio, but caused the accumulation of p62, which in turn activated autophagy to provide protection. Although our experiment did not find that bicyclol could change the expression of pro-autophagy protein Beclin-1, previous study has reported that Beclin-1 and Atg7 were up-regulated by bicyclol (Zhao et al., 2020). All these demonstrate that bicyclol can resist the progression of cholestasis liver injury through autophagy.

The p62-Keap1-Nrf2 pathway is part of the cellular antioxidant defense mechanism, in which p62 accumulates and competitively combines with Keap1 to activate Nrf2 (Komatsu et al., 2010). A recent study showed that activation of the p62-Keap1-Nrf2 pathway protects the ferroptosis in hepatocellular carcinoma cells (Sun et al., 2016). In our study, we found that the decrease of p62 led to the upregulation of Keap1, and Nrf2 was no significant changes. The accumulation of p62 caused by bicyclol treatment resulted in the degradation of Keap1 and activation of Nrf2. Nrf2 is one of the nuclear transcription factors, which provide protection against oxidative stress by regulating the expression of endogenous antioxidants. Genes regulated by Nrf2 are involved in the synthesis and regeneration of GSH, the expression of GST and GPx, the production of NADPH, and the expression of genes encoding iron chelation (Battino et al., 2018). In this study, we detected the mRNA levels of *Nrf2* and its downstream target genes *Nqo1*, *Hmox1*, and *Gclc*. Our results demonstrate that *Gclc* was not significantly different in animal experiments or cell experiments (data not shown). Heping *et al* reported that the mRNA and protein levels of GCLC, GCLM, and NRF2 all temporarily increased during BDL and then fell to baseline or below. These results suggested that either ursodeoxycholic acid or S-adenosylmethionine therapy alone can prevent the down-regulation of GCLC, GCLM, and NRF2 protein levels, while the combination of the two can increase these levels even higher (Yang et al., 2009). Other studies have shown that BDL leads to increased nuclear migration of NRF2, the expression of downstream target genes *Nqo1*, *Hmox1*, and *Gclm* is also up-regulated, and Vitamin A or baicalin intervention further increases the above performance (Wang et al., 2014; Shen et al., 2017). In our study, the mRNA level of *Nrf2* was not significantly up-regulated in the BDL group, while the levels of *Nqo1* and *Hmox1* were substantially up-regulated. Bicyclol not only up-regulated the mRNA level of *Nqo1* and *Hmox1*, but it also up-regulated the level of *Nrf2*. *In vitro* cell experiments, TCA only up-regulated the level of *Nrf2* mRNA without significant difference, while bicyclol up-regulated the mRNA and protein levels of *Nrf2* and *Nqo1*. So, bicyclol also improved liver damage caused by BDL by activating *Nrf2* and its downstream targets *Nqo1* and *Hmox1*, which is consistent with previous studies (Zhang et al., 2014; Zhao et al., 2020).

## Conclusion

These results indicate that bicyclol can improve liver damage caused by BDL in obstructive cholestasis. The bicyclol treatment promotes hydrophilization of bile acids, inhibits bile acid synthesis through Fxr/Cyp7a1, and promotes the excretion of bile acids through the Fxr/Bsep pathway. In addition, the fact that bicyclol treatment exerts its therapeutic effect through p62-Nrf2 anti-inflammatory and antioxidant pathways further the research on cholestasis liver injury.

**Institutional Review Board Statement:** The study was conducted according to the guidelines of the Declaration of Helsinki, and approved by the Institutional Review Board (or Ethics Committee) of Radiation Medicine Chinese Academy of Medical Sciences (IRM-DWLL-2018104, November 20, 2018).

## Data Availability

The raw data supporting the conclusions of this article will be made available by the authors, without undue reservation, to any qualified researcher.
